# Quality control of [^177^Lu]Lu-PSMA preparations using HPLC: effect of sample composition and ligand on recovery

**DOI:** 10.1186/s41181-022-00178-9

**Published:** 2022-09-27

**Authors:** Else A. Aalbersberg, Tammie T. Cao, Martine M. Geluk-Jonker, Jeroen J. M. A. Hendrikx

**Affiliations:** 1 Department of Nuclear Medicine, Netherlands Cancer Institute, Plesmanlaan 121, 1066 CX Amsterdam, The Netherlands; 2 Present Address: Department of Pharmacy and Pharmacology, Netherlands Cancer Institute, Plesmanlaan 121, 1066 CX Amsterdam, The Netherlands; 3 Department of Pharmacology, OLVG Locatie West, Jan Tooropstraat 164, 1061 AE Amsterdam, The Netherlands

**Keywords:** PSMA, [^177^Lu]Lu-PSMA-I&T, [^177^Lu]Lu-PSMA-617, HPLC, Column interaction

## Abstract

**Background:**

[^177^Lu]Lu-PSMA is used for the treatment of metastatic castration-resistant prostate cancer. For in-house productions, quality control methods are essential for ensuring product quality, and thus patient safety. During HPLC method development for quality control of [^177^Lu]Lu-PSMA-I&T, we noticed an unpredictable variability in peak area and height with replicate measurements. After a run, irremovable radioactivity was measured over the whole the length of the HPLC column, with slightly higher activity at the beginning and end of the column. The uniform distribution suggests that [^177^Lu]Lu-PSMA-I&T interacts with the column. As a result of the interaction, incomplete and variable recovery of injected activity was observed leading to the variability in peak area and height. Therefore the aim of this study was to (1) investigate the effect of sample composition on the interaction of [^177^Lu]Lu-PSMA-I&T to the HPLC column (measured as recovery, peak area, and peak height), and (2) to compare this with same concentrations of the well-known [^177^Lu]Lu-PSMA-617.

**Results:**

Sample composition significantly affects recovery of [^177^Lu]Lu-PSMA-I&T, leading to a change in peak area and height. Recovery was 24% when diluted with 0.1 mM octreotide, 38% with water, and increased to 95% when diluted with 0.7 mM unlabeled PSMA-I&T. Peak area and height decreased to 26% and 17% when diluted in octreotide and to 41% and 29% when diluted in water, compared to a dilution in PSMA-I&T. Further experiments showed that recovery (and consequently peak area and peak height) reached a plateau of > 99% at concentrations of 0.27 mM and higher. [^177^Lu]Lu-PSMA-617 also interacts with the HPLC column, leading to lower, but less variable, recovery (9%). The low recovery of [^177^Lu]Lu-PSMA-617 could not be prevented with addition of unlabeled PSMA-617.

**Conclusion:**

[^177^Lu]Lu-PSMA-I&T can undergo an irreversible binding with an HPLC column resulting in a decreased recovery. The recovery is can be highly dependent on sample composition. The addition of a surplus of unlabeled PSMA-I&T leads to an accurate analysis of [^177^Lu]Lu-PSMA-I&T.

## Introduction

Metastatic castration-resistant prostate cancer (mCRPC) has a poor prognosis, with a median survival just over one year (Aly et al. 2020). Treatment of mCRPC has recently been expanded to include [^177^Lu]Lu-PSMA. Prostate specific membrane antigen is a protein that is highly expressed on prostate cancer cells, but also has low to moderate expression in normal tissues such as salivary glands and liver (Afshar-Oromieh et al. 2013). [^177^Lu]Lu-PSMA binds specifically to this protein, delivering radiation to the tumor locally with the beta-emitting isotope Lutetium-177 while avoiding healthy tissue damage to organs at risk during external beam radiotherapy such as the rectum and bladder. In a large phase 3 study, [^177^Lu]Lu-PSMA-617 combined with standard care increased the median overall survival to 15.3 months, compared to 11.3 months in patients with standard care alone (Sartor et al. 2021).

Different ligands have been developed for [^177^Lu]Lu-PSMA radioligand therapy, with PSMA-617 and PSMA-I&T being the most commonly used and studied (Sadaghiani et al. 2021). For in-house productions of [^177^Lu]Lu-PSMA, PSMA-I&T is the ligand of choice since it is commercially available. Following an in-house production, an appropriate quality control must be performed to guarantee a safe product (Gillings et al. 2021). High-performance liquid chromatography (HPLC) coupled with a radio detector is the preferred semi-quantitative method for determining radiochemical purity of [^177^Lu]Lu-PSMA (Gillings et al. 2020). HPLC of radiolabeled PSMA-ligands (such as PSMA-I&T, PSMA-I&S, PSMA-I&F, PSMA-11, PSMA-1007, PSMA-617) is almost always performed with a reversed phase C18 column (generally with a pore size between 120 and 300 Å, a length of 150–250 mm, diameter of 3.0–4.6 mm, and a particle size of 3–5 µm, although many different brands are reported), with a gradient of acidified water and acetonitrile as the most common mobile phase (Migliari et al. 2017; Schottelius et al. 2019; Wang et al. 2018; Aalbersberg et al. 2020; Fuscaldi et al. 2014; Hooijman et al. 2021; Iudicello et al. 2021; Katzschmann et al. 2021; Ugur et al. 2021; European Pharmacopoeia 2021).

**Fig. 1:**
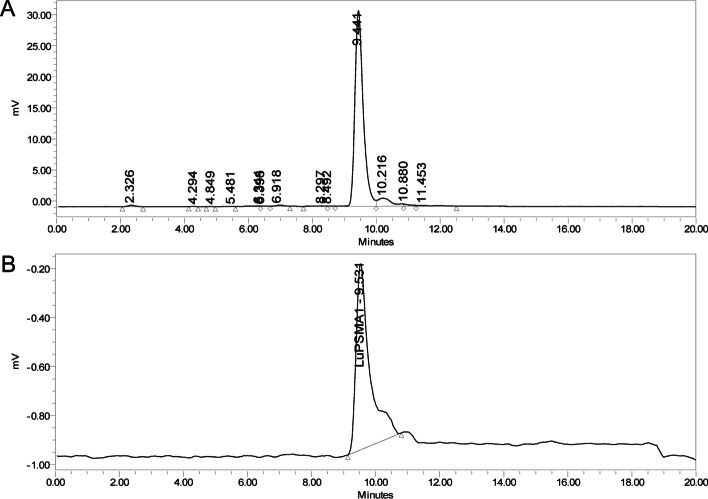
**A** Expected signal of [^177^Lu]Lu-PSMA-I&T on the radiodetector. **B** Unexpected low signal of [^177^Lu]Lu-PSMA-I&T on the radiodetector

At our laboratory an analytical HPLC method for determination of radiochemical purity of [^177^Lu]Lu-PSMA-I&T, similar to that of [^177^Lu]Lu-HA-DOTATATE (Andel et al. 2021), has been developed and validated according to EANM (Gillings et al. 2020), European Pharmacopoeia (2016), and EDQM (European Pharmacopoeia 2022) guidelines. Using the same column and mobile phase for both [^177^Lu]Lu-HA-DOTATATE and [^177^Lu]Lu-PSMA-I&T prevents having to clean, change, and equilibrate the HPLC column between different quality control samples which is time-consuming. With the use of this standard HPLC set-up for [^177^Lu]Lu-PSMA-I&T, we noticed an unpredictable variability in peak area and peak height between replicate measurements that occurred sporadically (Fig. 1). Based on preliminary tests, it seemed that the sample composition affected the interaction of [^177^Lu]Lu-PSMA-I&T to the column. Therefore the aim of this study was to (1) investigate the effect of sample composition on the interaction of [^177^Lu]Lu-PSMA-I&T to the HPLC column (measured as recovery, peak area, and peak height), and (2) to compare this with same concentrations of [^177^Lu]Lu-PSMA-617.

## Results

### Identifying the problem

Technical failure (e.g. variations in injection volume) during the measurements was ruled out and after yearly maintenance and requalification of the HPLC system a recovery screening was performed using a scintillation contamination monitor to determine remaining radioactivity in the system after each injection. Radioactivity was measured mainly at the HPLC column and limited radioactivity was measured in the tubing. Furthermore, batch effects of the column were excluded by using an identical column from a different batch. Additionally 2 different subtypes of the column (shielded vs. non-shielded) obtained similar results. Similarly, different mobile phases (based on methanol or ethanol), pH modifiers (ammonia, NaOH), and additional solvent (DMSO) were tested to prevent the interaction of PSMA-I&T with the HPLC column, but this also proved to no avail.

### Effect of HPLC sample composition on recovery of [^177^Lu]Lu-PSMA-I&T

**Fig. 2:**
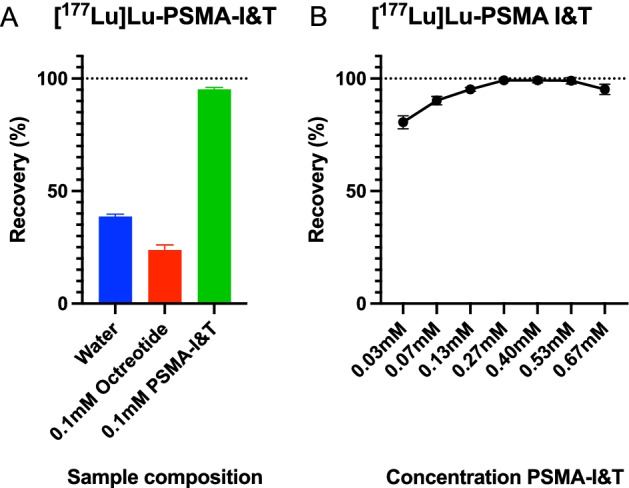
**A** Recovery (%) of [^177^Lu]Lu-PSMA-I&T diluted in 0.1 mM octreotide, water, or 0.7 mM PSMA-I&T. **B** Recovery (%) [^177^Lu]Lu-PSMA-I&T diluted in different concentrations of PSMA-I&T. Plotted data are mean and SD

The effect of HPLC sample composition was investigated by determination of the recovery after using multiple HPLC sample compositions. Sample composition had a statistically significant effect on recovery of [^177^Lu]Lu-PMSA-I&T as shown in Fig. 2A. The mean recovery of activity was 24 ± 2% of the injected activity when [^177^Lu]Lu-PMSA-I&T was diluted in 0.1 mM (0.1 mg/mL) octreotide, 38 ± 1% when diluted in water, and 95 ± 2% when diluted in 0.7 mM (1.0 mg/mL) PSMA-I&T (p < 0.0001).

### Effect of HPLC sample composition on peak area and height of [^177^Lu]Lu-PSMA-I&T

**Fig. 3:**
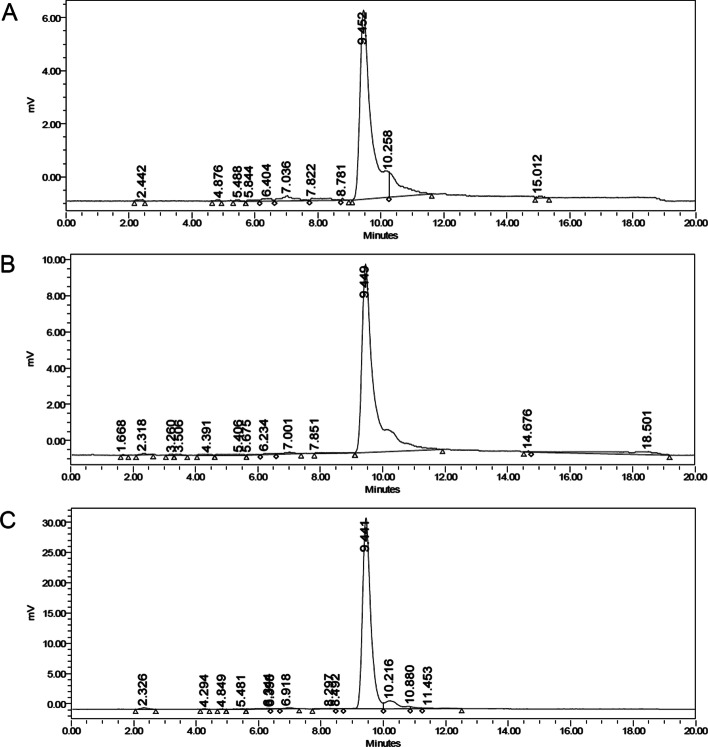
Typical radio-chromatograms after injection of 20 µL of [^177^Lu]Lu-PSMA-I&T dissolved in **A** 0.1 mM octreotide, **B** water, or **C** 0.7 mM PSMA-I&T. Note the differences in scale of the y-axis, which is adjusted to the height of the main peak

HPLC sample composition had not only a statistically significant effect on recovery, but also on peak area and height of [^177^Lu]Lu-PMSA-I&T and thus precision and accuracy of the assay. Peak area and height was decreased to 25.8 ± 2.7% and 17.1 ± 2.5% respectively when diluted in 0.1 mM octreotide compared to a dilution in 0.1 mM PSMA-I&T, and 41.7 ± 0.4% and 29.9 ± 0.4% when diluted in water compared to a dilution in 0.1 mM PSMA-I&T (p < 0.0001 for peak area, p < 0.0001 for peak height). Precision (measured as % relative standard deviation) was 8.7% for peak area and 11.5% for peak height with [^177^Lu]Lu-PMSA-I&T in 0.1 mM octreotide, 2.7% and 2.5% in water, and 2.0% and 3.3% in 0.1 mM PSMA-I&T. Typical chromatograms for each HPLC sample composition are shown in Fig. 3.

### Effect of ligand concentration on peak area and height of [^177^Lu]Lu-PSMA-I&T

Since adding 0.7 mM (1.0 mg/mL) of ligand to each sample increases the cost of the analysis, it is desirable to use the least amount while still producing optimal results. For [^177^Lu]Lu-PMSA-I&T, increasing the amount of ligand from 0.03 to 0.27 mM (0.05–0.4 mg/mL) increased peak area and height up (and thus recovery), while in the range of 0.27–0.7 mM ligand addition (0.4–1.0 mg/mL) a plateau is reached (Fig. 2B). Therefore the addition of 0.27 mM ligand is recommended for optimal recovery.

### Effect of HPLC sample composition on [^177^Lu]Lu-PSMA-617

**Fig. 4:**
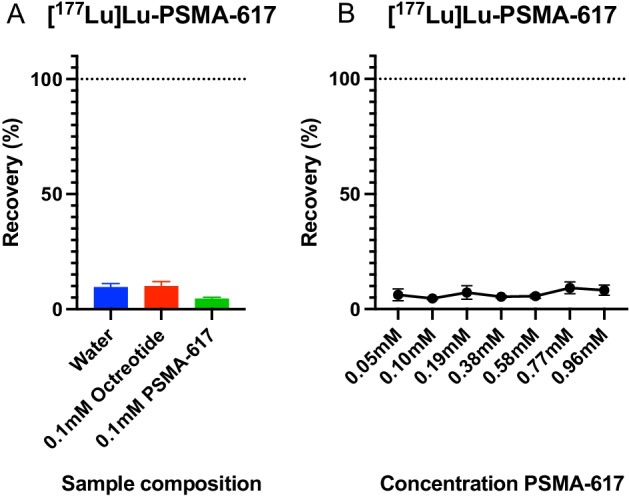
**A** Recovery (%) of [^177^Lu]Lu-PSMA-617 diluted in 0.1 mM octreotide, water, or 1.0 mM PSMA-617. **B** Recovery (%) [^177^Lu]Lu-PSMA-617 diluted in different concentrations of PSMA-617. Plotted data are mean and SD

For [^177^Lu]Lu-PMSA-617, HPLC sample composition did not lead to a change in recovery, peak area, or peak height. Recovery was 10 ± 2% when diluted in 0.1 mM octreotide, 9 ± 1% when diluted in water, and 5 ± 0% when diluted in 0.1 mM (0.1 mg/ml PSMA-617) (Fig. 4A). Peak area and height was not significantly different between the groups (p = 0.6187 for peak area, p = 0.9247 for peak height). Different ligand concentrations in the sample had also no effect on recovery of [^177^Lu]Lu-PMSA-617 (Fig. 4B).

## Discussion

We discovered variable responses during method development of an HPLC assay for QC of in-house preparation of Lu-PSMA. In this study we demonstrated that sample composition has a large impact on the recovery, peak height, and peak area and thus affected QC negatively.

Decreased recovery, and subsequently a decreased peak area or peak height, can negatively affect the outcome of quality control results. When the recovery decreases, there is a risk that smaller peaks (such as radiolysis products or unbound ^177^LuCl_3_) are below the detection limit of the system. This could lead to an incorrect overestimation of the radiochemical purity. Although we used a similar HPLC method (e.g. type of column, mobile phase, gradient, and run time) as described many times for different PSMA-based radiopharmaceuticals (Migliari et al. 2017; Schottelius et al. 2019; Wang et al. 2018; Aalbersberg et al. 2020; Fuscaldi et al. 2014; Hooijman et al. 2021; Iudicello et al. 2021; Katzschmann et al. 2021; Ugur et al. 2021; European Pharmacopoeia 2021), we experienced the importance of sample composition on recovery. Almost none of the previously published studies mention the sample composition or dilution of the HPLC sample, and only a few studies reported recovery. In our opinion, the importance of sample composition and recovery in method development and validation is underestimated and should be described in more detail in method reports to show method performance. This study reiterates the importance of determining recovery during HPLC method validation as precision and quantification limits were negatively impacted by a low recovery. Determination of recovery is relatively simple for radio-HPLC (either capturing the entire sample and measuring the activity or making use of a calibration line). The addition of recovery determination early in the process of HPLC method development when the expected peak height is unknown ensures that column interaction is detected early on and prevention strategies can be employed.

We encountered an irreversible interaction of [^177^Lu]Lu-PSMA-I&T with the HPLC column. Activity was present of the entire length of the column, and not for example solely on the column frits (Sadek et al. 1985). Carry-over into subsequent samples was limited and after washing and rinsing of the column, activity could still be measured over the entire length of the column. We hypothesized that the addition of a surplus of non-labeled PSMA-I&T (in this case ~ 250-fold) leads to a saturation of all available interaction sites for PSMA-I&T on the HPLC column leading to a complete recovery of simultaneously injected [^177^Lu]Lu-PSMA-I&T. Due to the high costs of ligands, maximizing the effect with minimal amount of ligand is desirable. In this study 0.27 mM (0.4 mg/ml) PSMA-I&T was found to be the optimal amount.

Other strategies to reduce the interaction of [^177^Lu]Lu-PSMA-I&T to the column could be the use of other solvents or other HPLC columns. The mobile phase used in this study (based on TFA, acetonitrile and water) is commonly used for various PSMA variants (Migliari et al. 2017; Schottelius et al. 2019; Wang et al. 2018; Aalbersberg et al. 2020; Fuscaldi et al. 2014; Hooijman et al. 2021; Iudicello et al. 2021; Katzschmann et al. 2021; Ugur et al. 2021; European Pharmacopoeia 2021). Different mobile phases, pH modifiers and solvents were evaluated but this did not prevent the interaction of PSMA-I&T with the HPLC column. Another solution could be using a different HPLC column. This could simply be a C18 column of a different brand, or alternatively a column that does not employ C18 or a core–shell column. The latter however might have pronounced effects on separation and retention.

Although both [^177^Lu]Lu-PSMA-I&T and [^177^Lu]Lu-PSMA-617 interact with the HPLC column, the addition of PSMA-I&T to [^177^Lu]Lu-PSMA-I&T increases the recovery of [^177^Lu]Lu-PSMA-I&T, the addition of PSMA-617 to [^177^Lu]Lu-PSMA-617 does not increase the recovery of [^177^Lu]Lu-PSMA-617. These ligands are identical in chelator (DOTA) and PSMA binding motif but differ in linker, suggesting that the linker might be responsible for the difference in behavior. However, since the HPLC assay for clinical [^177^Lu]Lu-PSMA-I&T could be used with the addition of extra ligand, determining the exact mode of interaction was beyond the scope of this research.

## Conclusion

[^177^Lu]Lu-PSMA-I&T can undergo an irreversible interaction with the C18 HPLC column. The addition of a surplus of PSMA-I&T saturates all interaction sites and leads to an accurate analysis of [^177^Lu]Lu-PSMA-I&T with a recovery of 97%. This effect of sample composition was not observed for [^177^Lu]Lu-PSMA-617. This study illustrates the importance of sample composition on method performance of [^177^Lu]Lu-PSMA-I&T assays and therefore we advocate for detailed description of sample composition in HPLC assays for quality control of radiopharmaceuticals.

## Materials and methods

### Reagents and chemicals

PSMA-I&T, PSMA-617, and the reagent and hardware kit for synthesis of ^177^Lu peptides (SC-05) were obtained from ABX (Radeberg, Germany), acetonitrile (> 99.9%) and water (Ultrapure LC/MS) from Biosolve (Dieuze, France), octreotide (0.1 mg/ml) from Sandoz (Holzkirchen, Germany), trifluoroacetic acid from Merck (Kenilworth, United States), 96% ethanol from SAHZ (Haarlem, the Netherlands), and [^177^Lu]LuCl_3_ (EndolucinBeta) from ITM Medical Isotopes GmbH (Munich, Germany).

### In-house production of [^177^Lu]Lu-PSMA-I&T and [^177^Lu]Lu-PSMA-617

[^177^Lu]Lu-PSMA-I&T and [^177^Lu]Lu-PSMA-617 was produced with a Scintomics GRP module (Scintomics, Fürstenfeldbruck, Germany) and reagent and hardware kit. In short, 4 mg 2,5-dihydroxybenzoic acid, 13 mg sodium ascorbate, and 31 mg sodium acetate-trihydrate are dissolved in 6 ml 0.04 M acetic acid. Together with 18 GBq [^177^Lu]LuCl_3_ solution, the acetic acid solution was added to 250 µg PSMA-I&T or 250 µg PSMA-617 freshly dissolved in 1 mL water and heated at 100 °C for 20 min. After sterile filtration through a 0.2 µm filter, the solution containing [^177^Lu]Lu-PSMA-I&T was diluted to 15 ml in NaCl/DTPA containing 8% ethanol.

### HPLC system and method

A Waters Acquity Arc system was equipped with a Waters 2998 photodiode array (PDA) detector (Milford, USA) and a Berthold Lb 514 Flowstar detector containing a BGO-X gamma measuring cell (Bad Wildbad, Germany). A Waters e-SAT/IN Module was used to transfer the analog signal of the radiodetector (mV) to a digital signal (counts). Eluent A consisted of 0.1% TFA in water and Eluent B was 0.1% TFA in acetonitrile. Chromatographic separation was achieved with a reversed phase Waters Shield C18 column with an inner diameter of 4.6 mm, length of 250 mm, and particle size of 5 µm. A starting eluent composition of 85% A/15% B was applied and decreased to 65% A/35% B over 16 min, followed by 4 min of isocratic 85% A/15% B, resulting in a total run length of 20 min. The injection volume was 20 µL. Radiodetection was at 20–1023 keV and UV detection at 220 nm. The method has been validated for the detection- and quantification limit, identification, carry-over, stability-indicating capacity, linearity, accuracy, precision, and robustness according to the current guidelines (Ugur et al. 2021; Andel et al. 2021) and standard operating procedures from our institute.

### Dilution of HPLC sample

Dilution of the sample is required in order to obtain a radioactivity concentration within the linear range of the radio detector without detector saturation. Due to the low concentration of PSMA-I&T in the sample, no signal can be obtained on the UV detector. Therefore the sample was originally diluted in octreotide, as a validated internal standard.

### Effect of HPLC sample composition on column interaction

To determine the effect of the sample composition on column interaction, [^177^Lu]Lu-PSMA-I&T was diluted 1:11 in 0.1 mM octreotide, water, or 0.1 mM PSMA-I&T and [^177^Lu]Lu-PSMA-617 was diluted 1:11 in octreotide 0.1 mM, water or 0.1 mM PSMA-617. Each sample was measured five times with HPLC and the recovery was determined.

To determine recovery, the area of all integrated peaks was used to calculate the activity in the sample, based on the calibration curve for ^177^Lu for the system (Katzschmann et al. 2021).

### Effect of solvent ligand concentration on column interaction

To determine the effect of ligand concentration on column interaction, [^177^Lu]Lu-PSMA-I&T was diluted in PSMA-I&T and [^177^Lu]Lu-PSMA-617 was diluted in PSMA-617 in the following concentrations: for PSMA I&T 0.03, 0.07, 0.13, 0.27, 0.40, 0.53 and 0.67 mM (0.05, 0.1, 0.2, 0.4, 0.6, 0.8, and 1.0 mg/ml, respectively) and for PMSA-617 0.05, 0.10, 0.19, 0.38, 0.58, 0.77, and 0.96 mM (0.05, 0.1, 0.2, 0.4, 0.6, 0.8, and 1 mg/ml, respectively). Each sample was measured five times with HPLC.

### Data analysis

Data analysis was performed in Excel (v14, Microsoft Corporation, Redmond, United States). All samples were corrected for decay and starting activity. Figures were created- and statistical analyses performed in GraphPad Prism (v7.03, GraphPad Software Inc, San Diego, United States). All data is presented as mean ± standard deviation, one-way ANOVA was used to test for significant differences between groups. When ANOVA showed significant differences between multiple groups, Tukey’s multiple comparisons test was performed to determine the significance between pairs.

## Data Availability

The datasets used and/or analyzed during the current study are available from the corresponding author on reasonable request.

## Abbreviations

ANOVA: Analysis of variance
DTPA: Diethylenetriaminepentaacetic acid
EANM: European association of nuclear medicine
EDQM: European directorate for the quality of medicines and healthcare
HPLC: High performance liquid chromatography
^177^Lu: Lutetium-177
mCRPC: Metastatic castration-resistant prostate cancer
NaCl: Sodium chloride
NaOH: Sodium hydroxide
PSMA: Prostate specific membrane antigen
TFA: Trifluoroacetic acid
UV: Ultraviolet
